# Response Mechanism for Surface Acoustic Wave Gas Sensors Based on Surface-Adsorption

**DOI:** 10.3390/s140406844

**Published:** 2014-04-16

**Authors:** Jiansheng Liu, Yanyan Lu

**Affiliations:** Institute of Acoustics, Chinese Academy of Sciences, Beijing 100190, China; E-Mail: luyanyan@mail.ioa.ac.cn

**Keywords:** surface acoustic wave (SAW), gas sensor, physical adsorption

## Abstract

A theoretical model is established to describe the response mechanism of surface acoustic wave (SAW) gas sensors based on physical adsorption on the detector surface. Wohljent's method is utilized to describe the relationship of sensor output (frequency shift of SAW oscillator) and the mass loaded on the detector surface. The Brunauer-Emmett-Teller (BET) formula and its improved form are introduced to depict the adsorption behavior of gas on the detector surface. By combining the two methods, we obtain a theoretical model for the response mechanism of SAW gas sensors. By using a commercial SAW gas chromatography (GC) analyzer, an experiment is performed to measure the frequency shifts caused by different concentration of dimethyl methylphosphonate (DMMP). The parameters in the model are given by fitting the experimental results and the theoretical curve agrees well with the experimental data.

## 1. Introduction

**Figure 1.**
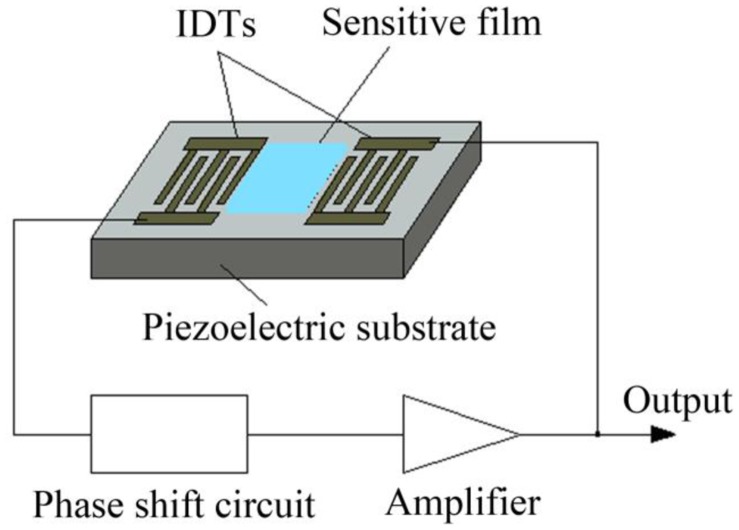
Schematic of SAW gas sensors.

Since White and Voltmer [1] excited surface acoustic waves (SAWs) by utilizing interdigital transducers (IDTs) deposited on a piezoelectric crystal surface, SAW technology has developed rapidly and is widely used in the telecommunication and other areas. Because the energy of SAWs is conserved near the surface of the piezoelectric substrate, a surface perturbation will lead to significant changes in surface acoustic wave properties such as propagation velocity, phase, attenuation and wave form. This characteristic can be used to develop acoustic sensors with good performance. Wohltjen and Dessy [2] first reported a chemical sensor for organic gas detection by coating a sensitive film on the surface of a SAW device. Since then a variety of SAW gas sensors have been developed for gas sensing [3–5]. The key unit of most of the reported sensors is a SAW oscillator (shown in Figure 1), which consists of a periphery circuit, a SAW detector and a sensitive film deposited on the detector surface. The sensitive film can strongly absorb a certain kind of gasses and almost dose not absorb other gasses; therefore we can obtain the content of the gas by measuring the change in oscillation frequency. The emphasis of this kind of sensor is to coat a sensitive film with high selectivity and high adsorption capacity. Such a SAW sensor or sensor matrix can only perceive one or several kind of gases; thus they are applicable to measure the content of some special gases [6–9]. In many areas, such as environmental monitoring, food security, explosive detection, there is a strong need for sensors which have a wide detecting range to monitor volatile organic or semi-volatile organic compounds (VOCs or SVOCs) [10–12]. To satisfy the demand, another kind of SAW sensor [13–17] was reported, in which a gas separation apparatus such as a gas chromatography (GC) column is set in front of the detector to separate and identify the tested multi-component gas. A multi-component gas can be separated and identified by their characteristic retention time in the GC column; thus SAW-GC sensors are available to detect VOCs and SVOCs in a wide range.

Many theoretical studies on SAW gas sensors have been reported. The basic theoretical model for SAW gas sensors is mainly Wohljent's method [18], which is based on the perturbation formula relating SAW velocity to film properties for the case of an acoustically thin film. Later Martin [19] expanded the method and his model is also applicable for the case of an acoustically thick film. The main problem they focused on is the changes in SAW device performance caused by a known layer on the device surface. If the quantity of the absorbed gas is known, the sensor output can be derived by using their theories. However, for a practical sensor, one must know the relationship between the output signal (changes in oscillation frequency of oscillators) and the measured gas properties, such as concentration and type. The type of gas can be identified by the selectivity of sensitive film or the characteristic retention time in GC column; therefore it is required to know the relationship between the adsorption amount and gas concentration.

Unfortunately, it is still very scarce the theoretical model for the relationship between the sensor output and the gas properties. Ricco [20] and Hietala [21] used SAW technology and commercial gas adsorption analyzer to measure the specific surface area and the pore size of porous film fabricated on SAW devices. Because commercial adsorption tools are adopted, the relationship between SAW sensor outputs and the gas concentration was not involved in their studies.

In this work, a theoretical model is presented for the response mechanism of SAW gas sensors with gas adsorption on the detector surface. In Section 2.1, the relationship between SAW sensor output (frequency or velocity shift) and the mass layer (the amount of absorbed gas) loaded on the surface of SAW detectors by using Wohljent's method is presented. In Section 2.2, the adsorption behavior of a gas on the surface of solids is analyzed; the BET formula and its improved form are introduced for describing the relationship between gas adsorption amounts and gas pressures which can be obtained from gas concentration by using the gas state equation. In Section 2.3, the relationship between SAW sensor outputs and gas pressures is brought out to describe the response mechanism for SAW gas sensors. A method is introduced to determine the model parameters by fitting the experimental data. In Section 3, an experiment is performed by using a SAW-GC gas sensor to verify the theoretical model. The frequency shifts caused by different concentration of DMMP is measured and the parameters in the model is given by fitting the experimental data. The theoretical curve is obtained and it agrees well with the experimental results.

## 2. Theoretical Analysis

### 2.1. Frequency Shift Caused by the Mass Loaded on a SAW Device Surface

The frequency shift of the SAW oscillator from the bare device is caused by the applied sensitive layer and the absorbed gas. In this work, only the change caused by the adsorption is included. Perturbation theory [18,19] and other methods such as the transfer matrix method are often used to calculate the frequency shift caused by the mass load. The transfer matrix method [22–24] is much more complicated although it has a more accurate solution. In this work, the amount of absorbed gas is tiny, the solution of Wohltjen's method, which is based the perturbation theory, has enough accuracy; therefore, Wohltjen's method is adopted due to its simple expression. We assume that the absorbed gas forms an isotropic, non-piezoelectric, non-conducting layer with a thickness of *h*, a density of *ρ* on the SAW detector surface. The change in oscillation frequency can be described by [18]:

(1) $$\Delta f=(k_{1}+k_{2})f_{0}^{2}\rho h-k_{2}f_{0}^{2}\rho h\frac{4\mu }{\nu_{0}^{2}}\left(\frac{\lambda +\mu }{\lambda +2\mu }\right)$$

where *k*_1_ and *k*_2_ are the coupling constants determined by the different displacement components of SAW in the substrate; *f*_0_ is the unperturbed oscillating frequency of the SAW oscillator, which is determined by the propagation velocity of SAW and the period of the IDTs fabricated on the surface of the piezoelectric substrate; *μ* and *λ* are the shear modulus and Lame constant of the layer; *ν*_0_ is the unperturbed velocity of SAW in the piezoelectric substrate. The first term in Equation (1) represents the frequency change caused by mass loading and the second term depends on the acoustic wave coupled into the layer. Because the layer formed by adsorbed gas is very thin, it can be seen as a simple mass load attached to the surface of the SAW device. Thus Equation (1) can be simplified as:

(2) $$\Delta f=(k_{1}+k_{2})f_{0}^{2}\sigma$$

where *σ* = *ρh* is the areal density of the layer formed by the adsorbed gas. Equation (2) means that the sensor output is proportional to the quantity of the mass loaded on the surface of a SAW device, and it is the theoretical basis for the detection of SAW sensors.

### 2.2. Adsorption Mechanism of Gas on the Surface of Solids

Due to intermolecular forces or chemical bonding force, when a gas contacts with the surface of a solid, part of the gas molecules will stay on the solid surface for some time. This phenomenon is called adsorption. The adsorption generated by the intermolecular force is a physical adsorption; the adsorption generated by chemical bonding force is a chemical adsorption. During the process of chemical adsorptions, chemical reactions will produce new molecules from the molecules of absorbed gas and the molecules in the solid surface, which means that the absorbed gas will be changed into a new material. Since chemical bonds are much stronger than molecular diffusions, chemical adsorptions are almost irreversible at room temperature and pressure, which means that no desorption will happen when the gas leaves. Thus, the sensor based on chemical adsorption is unrepeatable. Most of SAW gas sensors are required to be repeatable, so the adsorption is a physical one. Among all theoretical models for physical adsorption, the BET formula proposed by Brunauer, Emmett and Teller in 1938 [25] is the most commonly used method:

(3) $$\frac{V}{V_{1}}=\frac{cP}{\left(P_{0}-P)[1+(c-1)P/P_{0}\right]}$$

Where *V* is the gas volume occupied by the gas molecules adsorbed on the solid surface; *V*_1_ is the volume of the saturated monomolecular layer adsorption, which means the required gas volume for covering the solid surface with a single molecular layer; *P* is the gas pressure; *P*_0_ is the saturated vapor pressure at the adsorption temperature; *c* is an adsorption characteristic parameter, which is decided by the temperature, the average adsorption heat of the first monolayer and the gas liquefaction heat.

According to the equation of state of an ideal gas, we can get the gas pressure:

(4) $$P=\frac{\rho_{C}}{M}RT$$

where *ρ_C_* is the mass quantity of the test gas within a unit volume; *M* is the mass of per mole gas; *R* is the ideal gas constant; *T* is the adsorption temperature in the thermodynamic temperature scale. Equation (4) indicates that the gas pressure is proportional to its mass volume concentration.

Equation (3) is the well-known two-parameter BET equation; its best suitable interval of relative gas pressure is between 0.05 and 0.35. For convenience, we use *x* to represent the relative pressure *P*/*P*_0_ in this work. When *x* exceeds 0.35, the theoretical adsorption values will be higher than the experimental results. In order to obtain a larger suitable scope, many researchers try to improve the BET equation. In this study, a BET equation improved by Anderson [26] and Brunauer [27] is adopted:

(5) $$\frac{V}{V_{1}}=\frac{\text{ckx}}{\left(1-kx)[1+(c-1)kx\right]}$$

where *k* is an adjustable constant decided by the nature of the adsorption system. By selecting the appropriate value of *k*, we can get the theoretical results consistent with the experimental data at *x* of 0.05–0.9.

### 2.3. The Response Mechanism for SAW Gas Sensors Based on Surface-Adsorption

Obviously, the mass of the gas adsorbed in the surface of a SAW device is proportional to the gas volume occupied by the adsorbed gas molecules. Assuming that *σ*_1_ is the areal density with a monomolecular layer of adsorbed gas on the SAW detector surface, through Equation (2) we can get:

(6) $$\frac{\Delta f}{\Delta f_{1}}=\frac{\sigma }{\sigma_{1}}=\frac{V}{V_{1}}$$

where Δ*f*_1_ is the frequency offset of the SAW oscillator caused by a monomolecular layer covering the detector surface. Substituting Equation (6) into (5), we can get:

(7) $$\frac{\Delta f}{\Delta f_{1}}=\frac{\text{ckx}}{\left(1-kx)[1+(c-1)kx\right]}$$

Equation (7) expresses the relationship between the oscillation frequency shift and gas pressure (decided by the gas concentration and temperature), which is the theoretical model of the response mechanism for a SAW gas sensor with gas absorbed on the device surface directly.

The parameters of *Δf*_1_ and *c* in Equation (7) can be determined through fitting experimental data. Another form of Equation (7) is:

(8) $$\frac{kx}{\Delta f(1-kx)}=\frac{1}{\Delta f_{1}c}+\frac{\left(c-1\right)}{\Delta f_{1}c}kx$$

Equation (8) can be considered as a linear function of *kx*/[Δ*f* (1−*kx*)] with respect to *kx*. The slope *a* and the intercept *b* of the line can be gotten by linear fitting of the experimental data; then we can get Δ*f*_1_, the oscillation frequency shift caused by a monomolecular layer of adsorbed gas, and *c*, the adsorption characteristic parameter:

(9) $$\{\begin{array}{c} \Delta f_{1}={\left(a+b\right)}^{-1}\\ c=1+a/b \end{array}$$

## 3. Experimental Verification

**Figure 2.**
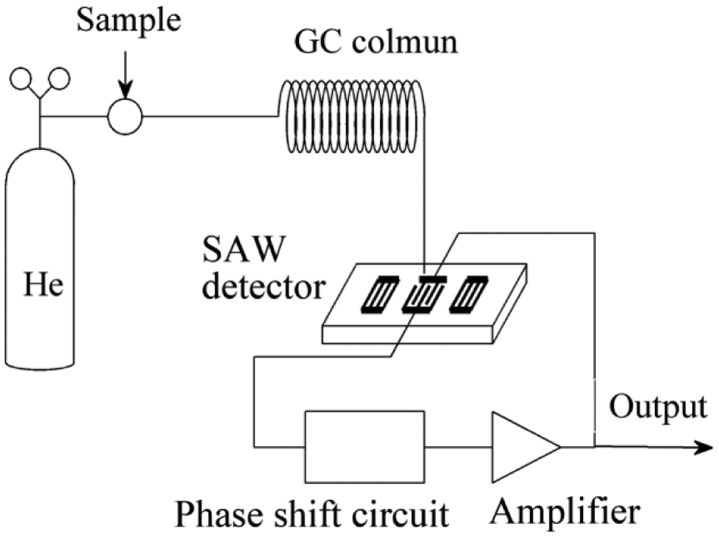
Schematic of the SAW-GC analyzer used in the experiment.

To verify the theoretical model, an experiment was performed by using an ultra-fast SAW-GC analyzer with a type of zNose 4200, produced by Electronic Sensor Technology (EST, Newbury Park, CA, USA). As shown in Figure 2, the analyzer is mainly composed of an injection unit, a separation and identification unit, and a detection unit. The injection unit consists of a carrier gas and a sampling part; the separation and identification unit of a programmable heated GC column; the detection unit of a SAW detector and an oscillating circuit. The carrier gas is helium which can pass through the GC column freely. When a multi-component gas is sampled, it will be carried through the GC column. Because each component has its own unique passage rate, the various gas components are separated and identified by their characteristic retention times in the GC column. When reaching the surface of the SAW detector, each gas component is absorbed in the surface of the detector and results in a perturbation on the propagation velocity of SAW, which leads to a frequency shift of the oscillation circuit; the content of each component gas can be obtained by measuring the frequency shift. The detector is a SAW resonator fabricated on ST-cut X-propagate quartz (its coupling constants *k*_1_ = −8.93 × 10^−8^ s·m^2^/kg and *k*_1_ = −3.86 × 10^−8^ s·m^2^/kg) and the operation frequency of the resonator is 500 MHz.

In our experiment, dimethyl methylphosphonate (DMMP) is input via the sample inlet; helium carries DMMP through the GC column and reaches the surface of the SAW detector. The temperature of the SAW detector is controlled at 25 °C. The gas concentration can be adjusted by changing the injection volume of DMMP and the corresponding frequency shift of the oscillation circuit is measured as the sensor output. The saturated vapor pressure *P*_0_ is the vapor pressure when the vapor is in equilibrium with the liquid at a certain temperature. A substance has different *P*_0_ at different temperatures, and *P*_0_ increases as the temperature increases. The saturated vapor pressure can be calculated by using the Antoine equation:

(10) $$\ln(P_{0})=A-\frac{B}{C+T}$$

where *P*_0_ is the saturated vapor pressure; *A*, *B* and *C* are empirical Antoine constants; *T* is the thermodynamic temperature. If *P*_0_ in Pascal, the Antoine constants of DMMP [28] are *A* = 22.319, *B* = 4,344.0 and *C* = −51.7. In this work, the temperature of the SAW detector is controlled at 25 °C (*T* = 298.15 K), substituting these parameters into Equation (10) we can get *P*_0_ = 111 Pa.

**Figure 3.**
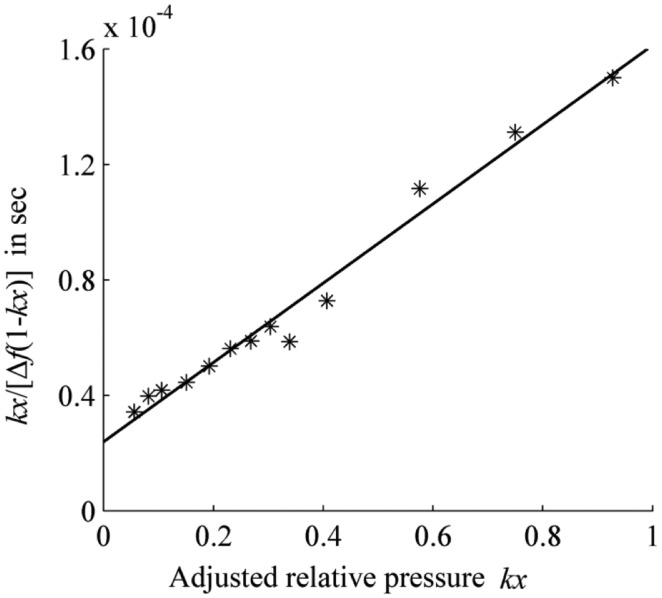
Linear fitting of *kx*/[Δ*f*(1 − *kx*)] *versus* adjusted relative pressure *kx* at the SAW detector temperature of 25 °C. Solid line: the fitting line; stars: the measured data.

Figure 3 shows a linear fit according to Equation (8) and the measured results; the adjustment factor *k* of the relative pressure is set as 1.35. The slope *a* of the fitting line is 1.37 × 10^−4^ and the intercept *b* is 2.39 × 10^−5^. Substituting these values into Equation (9), we obtain the Δ*f*_1_ of 6.20 *k*Hz and the characteristic adsorption constant *c* of 6.74. It should be noted that the adjustment factor *k* is less than 1 in Anderson's article, which is explained as the adsorption heat of the layers above the first layer is less than the heat of liquefaction. In this work, the adjustment factor *k* is greater than 1; the reason for this difference is perhaps that the adsorption heat of the layers above the first layer is more than the heat of liquefaction.

**Figure 4.**
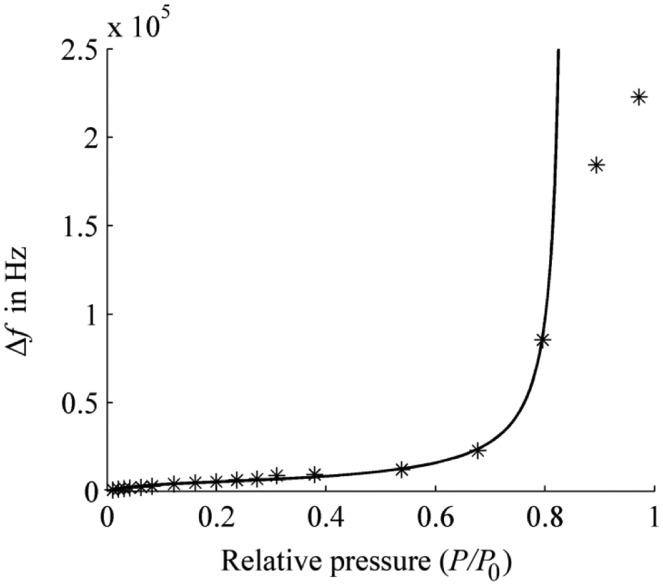
The oscillation frequency shift Δ*f versus* the relative pressure *P*/*P*_0_ at the detector temperature of 25 °C. Solid line: the calculated curve; stars: the measured data.

The theoretical and measured oscillation frequency shifts are compared in Figure 4. The theoretical sensor outputs are calculated by using Equation (7) and the parameters are listed in the previous paragraph. When the relative gas pressure is less than 0.8 (*kx* < 1), the theoretical curve has a wonderful agreement with the measured data.

Especially when *x* < 0.6, the sensor has a good detection performance with the frequency shifts proportional to the gas pressure (concentration) substantially. When the relative gas pressure exceeds 0.8 (*kx* close or more than 1), the theoretical predicted curves is obviously above on the experimental data. The reason for this is the assumption that at *kx* = 1 an infinite number of gas molecular layers are adsorbed in the BET equation; but in reality it is only a limited number of layers.

## 4. Conclusions

In this work, a theoretical model is put forth to describe the response mechanism for SAW gas sensors based on physical adsorption on the detector surface. By combining Wohljent's method and the modified BET formula, we obtain a theoretical model which describes the relationship of the sensor output (frequency shift of SAW oscillator) and the gas pressure (determined by the gas concentration and temperature). By using a commercial zNose 4200 SAW-GC gas sensor, an experiment is performed to measure the frequency shifts caused by different concentration of DMMP. The parameters in the theoretical model are given by fitting the experimental data and the theoretical curve of the sensor output is compared with the experimental data. When the relative gas pressure is less than 0.8, the theoretical curve agrees well with the experimental results. The model developed in this work and the discussions on the adsorption behavior of gas on the SAW detector surface will benefit attempts to achieve a SAW gas sensor with better performance.
